# Towards Viral Genome Annotation Standards, Report from the 2010 NCBI Annotation Workshop

**DOI:** 10.3390/v2102258

**Published:** 2010-10-13

**Authors:** James Rodney Brister, Yiming Bao, Carla Kuiken, Elliot J. Lefkowitz, Philippe Le Mercier, Raphael Leplae, Ramana Madupu, Richard H. Scheuermann, Seth Schobel, Donald Seto, Susmita Shrivastava, Peter Sterk, Qiandong Zeng, William Klimke, Tatiana Tatusova

**Affiliations:** 1 National Center for Biotechnology Information, National Library of Medicine, National Institutes of Health, Bethesda, MD 20892, USA; E-Mails: bao@ncbi.nlm.nih.gov (Y.B.); klimke@ncbi.nlm.nih.gov (W.K.); tatiana@ncbi.nlm.nih.gov (T.T.); 2 Los Alamos National Laboratory, Los Alamos, NM 87545, USA; E-Mail: kuiken@lanl.gov; 3 Department of Microbiology, University of Alabama at Birmingham, Birmingham, AL 35222, USA; E-Mail: elliotl@uab.edu; 4 Swiss Institute of Bioinformatics, 1211 Genève 4, Switzerland; E-Mail: philippe.lemercier@isb-sib.ch; 5 Laboratoire de Bioinformatique des Génomes et des Réseaux, Université Libre de Bruxelles, B-1050 Bruxelles, Belgium; E-Mail: raphael@bigre.ulb.ac.be; 6 J. Craig Venter Institute, Rockville, MD 20850, USA; E-Mails: rmadupu@jcvi.org (R.M.); sschobel@jcvi.org (S.S.); sshrivastava@jcvi.org (S.S.); 7 Department of Pathology and Division of Biomedical Informatics, University of Texas Southwestern Medical Center, Dallas, TX 75390, USA; E-Mail: richard.scheuermann@utsouthwestern.edu; 8 Department of Bioinformatics and Computational Biology, George Mason University, Manassas, VA 20110, USA; E-Mail: dseto@gmu.edu; 9 Wellcome Trust Sanger Institute, Wellcome Trust Genome Campus, Cambridge CB10 1SA, UK; E-Mail: ps8@sanger.ac.uk; 10 Broad Institute, Cambridge, MA 02141, USA; E-Mail: qzeng@broadinstitute.org

**Keywords:** virus, genome, annotation

## Abstract

Improvements in DNA sequencing technologies portend a new era in virology and could possibly lead to a giant leap in our understanding of viral evolution and ecology. Yet, as viral genome sequences begin to fill the world’s biological databases, it is critically important to recognize that the scientific promise of this era is dependent on consistent and comprehensive genome annotation. With this in mind, the NCBI Genome Annotation Workshop recently hosted a study group tasked with developing sequence, function, and metadata annotation standards for viral genomes. This report describes the issues involved in viral genome annotation and reviews policy recommendations presented at the NCBI Annotation Workshop.

## 1. Introduction

**Figure 1:**
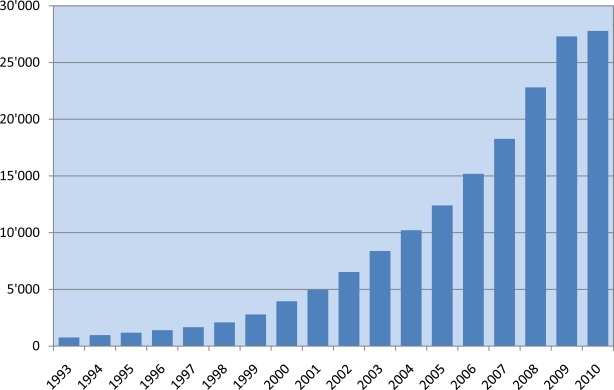
Cumulative number of validated full-length virus genome sequence records deposited in GenBank from 1993 to 2010 ^1,2^ ^1^ Only GenBank sequence records validated by RefSeq curators as full-length genomes are included. ^2^ Individual viral segments are included in tabulations, not complete constellations.

Spurred by increasingly inexpensive technologies, the pace of virus genome sequencing has risen sharply in the past decade (see Figure 1). Currently, there are 27,091 full-length virus genomes deposited in GenBank (see Table 1), and these are distributed among 2,500 distinct viral taxonomic groups [1]. Impressive as this is, the number of sequenced viral genomes is expected to grow rapidly over the foreseeable future as genome sequencing methodologies are utilized in viral discovery and surveillance efforts.

Virus genome sequencing efforts should illuminate a number of fundamental topics in biology, including genome evolution, interactions between hosts and parasites, and relationships between viruses and disease. However, it is important to note that the scientific significance of a given genome is dependent on identifying functional sequence features and placing them in a biological context. In practice, this means annotating the genome sequence with genes, open reading frames (ORFs), regulatory elements, and other genetic features, as well as providing “metadata” about the collection and characterization of the sequence.

Much of the virus genome sequence data is deposited in one of the public databases that together comprise the International Nucleotide Sequence Database Collaboration (INSDC), the DNA Database of Japan (DDBJ) [2], the European Nucleotide Archive (ENA) [3], and GenBank [4]. All of these databases are archival, and as such, the annotation of sequences submitted to them is primarily the responsibility of the submitter. So it is up to the scientific community itself to craft virus genome annotation standards and to promote their use by sequence submitters.

In April 2010, the National Center for Biotechnology (NCBI) co-sponsored a workshop with the J. Craig Venter Institute (JCVI) to discuss genome annotation issues—the third such meeting—which for the first time included a working group focused on virus genome annotation issues. This working group brought together a diverse group of individuals from the world’s nucleotide sequence databases and sequencing centers in the hopes of establishing a cohesive set of universally accepted standards for the annotation of virus genomes. This paper will outline the findings of this Virus Genome Working Group and highlight steps being taken to implement virus genome annotation standards.

## 2. Results and Discussion

### 2.1. Viral Genome Sequence Annotation Minimal Standards

A variety of computational gene prediction programs are used to functionally annotate viral genomes including GeneMarkS [5], Glimmer3 [6], and Zcurve [7,8]. These programs build gene models based on sequence signals (e.g., ribosomal binding sites, start codons, and stop codons) and statistical information within nucleotide sequences. Typically, these intrinsic methods are enriched with extrinsic sequence comparisons to reference genomes, proteins, and peptide domains, and most current annotation pipelines incorporate both intrinsic and extrinsic methodologies [9,10].

Unlike the cellular hosts they infect, viral genomes are composed of a variety of nucleic acid topologies, DNA and RNA, both single and double-stranded. There is also great variability in genome length, from Hepatitis delta virus (1,682 nt) to *Acanthamoeba polyphaga mimivirus* (1,181,404 nt), and in nucleotide composition, from less than 30% GC content to more than 65%. The physical variation among viral genomes is reflected by the wide expanse of gene structures, which can include overlapping ORFs, alternatively spliced transcripts, ribosomal slippage sites, and polyproteins that give rise to multiple mature peptides through post-translational proteolytic cleavage.

Physical sequence factors influence intrinsic computational gene prediction methods, and as they vary so does the accuracy of any given computational tool [10]. These core differences between viral genomes preclude the use of a single computational annotation methodology for all viruses. Rather, accurate annotation often requires the use of specialized tools designed for a specific family of viruses. The Virus Genome Working Group strives to facilitate the development of accurate annotation tools by bringing together teams of virologists, computational biologists, and computer scientists and to make these tools available to the scientific community at large.

### 2.2. Transfer of Genome Annotation from Public Resources

When a nucleotide record is deposited into DDBJ, ENA, or GenBank, the annotated coding sequences are used to create protein records. These in turn are disseminated to the various protein databases where they are available through BLAST searches and can be used as references for the annotation of still more genomes. This is a critical point, and it is important to realize that any errors in annotation can be propagated to other genomes. In other words, bad annotation begets more bad annotation. With this in mind, the common goal of all genome annotation endeavors must be accuracy.

Even in cases when competent computational annotation tools are available, some features like mature peptide cleavage sites and ribosomal slippage sites are difficult to predict with computational methods. One approach is to compare the newly sequenced genome to experimentally validated reference protein sets. While it seems unlikely that the protein coding regions of all viruses will be experimentally determined, in many cases there may be viruses within a family for which there is, or will be, detailed biochemical information. These so-called reference genomes can then be used as templates, providing invaluable references for the annotation of other, related genomes.

Several resources have been created to aid in the functional annotation of protein records. Annotated proteins are picked up by public resources such as PFAM [11] and Protein Clusters [12], where the translated sequences are aligned and grouped into clusters of homologous proteins. These clusters can then be curated en masse, allowing well-characterized proteins to seed the annotation of less well characterized ones. This is a particularly strong technique for ascribing biological function and names to conserved proteins. Protein clusters are also data containers aggregating many types of information related to the constituent proteins, such as structures, literature references, and other database resources, and linking it to all proteins within a given cluster.

### 2.3. Integration of Experimental Evidence into Genome Annotation

Given that public resources exist to transfer biological information from one protein to other related proteins, the problem is how to seed these resources with well-annotated proteins. There was a unanimous consensus among the Virus Working Group members that the best annotation is derived from experimental data. This clear fact emphasizes the role of sequence records as living documents that must be periodically updated with the latest experimental data. Though publications may remain the currency of research, the increasing importance of genomics portends a world wherein sequence records are the central source of biological information and BLAST homology searches are the primary entry portal to discovery.

The focus on experimentally validated data underscores the necessity of extending biochemical and genetic experimentation throughout the viral universe. While disease associations or industrial applications of some viruses will inevitably drive their molecular characterization, the genomes of many viruses will remain poorly described without focused experimental efforts. Similar calls to action have been made with reference to bacterial genomes culminating in the COMputational BRidge to Experiments (COMBREX) effort [13], which attempts to fund “small science” efforts to biochemically characterize conserved, but otherwise hypothetical proteins. The Virus Working Group members embraced such efforts and saw clear rationale for extending these approaches to viral proteins.

Conceptually, the updating of existing sequence records with new experimental data and ever-improving annotations seems fairly straightforward, at least once such data exists. The current infrastructure allows some updates, and requests can be sent directly to RefSeq [14] or GeneRIFs [15]. Yet, the curation process can require a great deal of time and effort and typically yields little, if any, scientific currency, *i.e.* publications, so even this seemingly mundane task is tasking. However, if the scientific community is serious about having up-to-date, experimentally validated sequence records, then there needs to be mechanisms for eliciting involvement of researchers in annotation efforts and rewarding them for their work [16]. Such a paradigm shift may seem unrealistic today, but without funding and reward opportunities tuned toward genome annotation, the term “conserved hypothetical protein” will continue to plague BLAST searches.

### 2.4. Protein Naming

Accurate functional annotation must be accompanied by unambiguous naming of genome features. This is particularly true for protein names. Discrepancies in protein names arise for many reasons, including historical usage, evolving experimental data, and lack of clear naming guidelines. Unfortunately this lack of cohesion can lead to confusion and outright inaccuracies. Even under the best circumstances, protein naming standards are not identical across the viral research community, and there is a need to consolidate naming standards.

There was a consensus among workshop attendees that all databases should use the same protein names on otherwise identical records. These should be functional protein names like those defined by UniProtKB/Swiss-Prot [17] protein naming guidelines, but traditional and historical names should be linked to the protein as alternative names in order to maintain consistency with previous literature. Moreover, these naming efforts must build on an open architecture that can be shared across all databases and still leaves room for more extended feature descriptions.

Gene Ontology (GO) [18,19] terms provide a controlled vocabulary for describing gene products and can provide information about multiple activities not revealed by a single name. Since GO terms can be assigned with experimental evidence codes linked to a PubMed unique IDentifier (PMID), they further support experimentally validated annotation [20]. Although these terms are not currently included in GenBank records, there was a consensus that GO terms offer unique information and should be better integrated into database resources where possible. Of course, none of this comes for free, and though the GO project requires sustained participation by members of the scientific community, the potential reward is a deep, standardized annotation vocabulary, consistent across species and databases.

Though NCBI and UniProt/Swiss-Prot have already begun the effort to harmonize protein naming, a variety of questions remain regarding the implementation of this goal. Uniprot protein records support multi layered naming approaches and NCBI’s Protein Clusters includes links to these records, but it is not clear how individual GenBank records can be linked to various layers of data like GO terms, evidence codes, and alternative protein names. Furthermore, all universal naming efforts need some sort of a clearinghouse where information can be updated and reviewed. This is particularly important to efforts that include multiple government databases where “wiki-styled” models may not be appropriate. As of yet, the policies and procedures necessary to facilitate multi-source curation await development.

### 2.5. Viral Genome Classification

One long-standing example of a multinational effort to develop universal virus standards is the International Committee on the Taxonomy of Viruses (ICTV) [21]. The ICTV is an independent body specifically engaged in the creation and validation of viral classification standards, which are universally accepted by the DDBJ, ENA, and GenBank. As might be expected, the dawn of the genomics era has radically changed approaches to viral taxonomy. Where in the past viruses could only be characterized by restriction enzyme digests, electron micrographs, and immunohistochemistry, the accessibility of genome sequencing allows in depth sequence analysis and comparison for an ever increasing number of newly discovered viral isolates.

Workshop attendees generally agreed that it is important for ICTV to pursue a policy to further integrate computer tools into viral classification standards. Specifically, the classification criteria and tools used by each ICTV study section should be transparent and published on the web, so that individual researchers and database curators have easily accessible reference. In cases where non-computational criteria are used for viral classification, it was proposed that descriptions of the standard operating procedures (SOPs) be integrated into the GenBank record. For example, when trans-complementation assays are used to determine species, this should be documented in the sequence record. This is especially important when sequences are not published, as there is no paper trail documenting the classification.

Members of the Virus Working Group have drafted a proposal to the ICTV, which argues for increased usage of computer based tools in taxonomical assignments. We are aware that this proposal is just a start to a longer process where individual computational tools are developed and assessed. This proposal highlights the use of the PAirwise Sequence Comparison tool (PASC) [22] in viral classification. Obviously, given the genetic breadth of the viral world, it will not be possible to identify a single classification protocol that works equally well for all viruses. Large dsDNA viruses will require different approaches to smaller ssRNA viruses and specific criteria and tools will need to be developed for individual classes of viruses. Rather, the key element is to move towards more transparent classification standards that use freely available, web hosted computational tools.

### 2.6. Viral Genome Metadata Minimal Standards

With a newly sequenced viral genome in hand and accurately annotated, the next step is to place the genome into a biological context. This requires more than just the genome sequence, and only when information about isolation source, geographic location, and date are included does a viral genome sequence become something more: a sample isolated in evolutionary time and environmental context, which can be compared to others, allowing inferences between sequence, host, chronology, and geography. Unfortunately, there are few adopted standards for the inclusion of these metadata in viral genome sequence records, often rendering the sequence unusable for deep analysis. This problem will only get worse as the shine of genome sequencing fades, and the INDC databases begin to fill with otherwise unpublished genome sequences. With no literature reference, metadata will be paramount to the scientific utility of these genomes.

The debate over viral genome metadata is not so much centered on what descriptors should be included with viral genome records, as it is fairly easy to agree on what types of metadata are important. Descriptors like collection source, host(s), collection date, and geographic identifier (e.g., isolation country or latitude and longitude coordinates), are absolutely essential to understanding the biological context of the viral genome, as are host-specific metadata like disease severity. Yet, getting these into genome records is difficult at best. This is particularly frustrating given the various efforts to design universal naming schemes in which metadata are included in the isolate name. The emphasis here is misplaced, as the rationale behind any specific naming scheme may not stand the test of time. Yet, the metadata is a constant, and as long as the relevant metadata is included in every genome record, any naming format will be supported.

To ensure community wide acceptance and usage, any attempt at virus genome metadata standards must include a highly visible outreach effort. With this in mind, a clear consensus emerged within the Working Group to join the previously initiated minimum information about a sequence (MIGS) [23] specification effort within the Genomic Standards Consortium (GSC) [24]. Our goal is to build upon the existing MIGS reference metadata checklist of standards [23] and foster their use within our respective institutions. This endeavor will require the collection of metadata terms, using existing ontologies (e.g., the Infectious Disease Ontology) and submitting them to the GSC for inclusion in reference checklists. It will also include mapping metadata to GenBank qualifiers and enumerating guidelines for appropriate use of structured comments when necessary.

## 3. Experimental Section

The data in Figure 1 and Table 1 were obtained by querying the RefSeq internal database for the number of reference sequences and genome neighbors (GenBank records validated as full-length genomes by RefSeq curators) belonging to specific taxonomic groups. The query was conducted September 16, 2010. RefSeq records and the corresponding GenBank record from which they were created were counted only once, not twice. The number of virus genome records present in past years was determined by GenBank submission date.

## 4. Conclusions

The first meeting of the NCBI Virus Genome Annotation Working Group revealed a number of persistent problems that must be addressed. Since genome annotation is actually the sum of a number of processes and touches on diverse computational and biological issues, it is unlikely that a single rigid organization can provide the necessary framework to advance genome standards and policies. Hence, it seems more reasonable for the Working Group to act as an umbrella organization, identifying issues and putting together smaller groups of experts to tackle them. To maintain a mandate for such activities, it is critical that Working Group activities remain transparent and open to comment and participation by concerned individuals.

The ultimate goal of the Working Group is to facilitate knowledge sharing between the various scientific organizations, journals, and individual researchers, develop collective policies, and find ways to implement them. Perhaps one of the easiest ways to promote these activities is to publish annotation resources on the web. A subgroup is now identifying currently available virus web resources, such as annotation pipelines and other computational tools, and the initial plan is to aggregate links to these on a single web page. In the future this web resource could be expanded to include viral genome submission guidelines, controlled vocabulary lists and ontologies, and other useful information. The idea here is to create a one stop portal, clearly visible to researchers and reviewers and designed to support the implementation of genome annotation standards.

Uniform standards across geography and databases will require specialized resources for communication and knowledge sharing between those actively engaged in genome curation. Such resources should include clearinghouses where viral genome curators from NCBI, EBI, DDBJ, UniProtKB//Swiss-Prot, and others can update protein names, revise experimentally derived annotation and exchange information. Though no specific ideas have been put forth, one model of annotation data exchange is the Wellcome Trust Sanger Institute Distributed Annotation System (DAS) [25]. Other resources will be necessary to verify taxonomic classification and naming guidelines, and these will need to be developed in concert with the ICTV and other virus specific groups.

Though curators can provide some level of support, successful implementation of virus annotation standards will require community involvement. Ideas that sound simple in the abstract can be difficult to implement both in scale and process, and it is important that members from all strata of the scientific community participate. A perfect example is a long heralded project to create “gold standard” viral genome records replete with updated experimental data that can be used to seed the annotation of less characterized genomes. This activity requires first the identification of suitable genomes, then the assembly of expert panels to curate these records, and finally the validation of curation efforts to make sure that they conform to accepted standards. Hence, to bring this seemingly simple idea to fruition will require cooperation between database curators, bioinformatics specialists, and virology experts, not to mention the constituent databases and organizations like the ICTV.

The Virus Genome Annotation Working Group will remain a functional entity as part of the NCBI Prokaryotic Annotation Workshop (NCBIPAW). NCBIPAW maintains a Google Group where annotation issues can be discussed and ideas exchanged. Information about NCBIPAW meetings and other activities are publicly available at www.ncbi.nlm.nih.gov/genomes/AnnotationWorkshop.html.

## Figures and Tables

**Table 1:** Total number of full-length virus genome sequence records in GenBank.

**Virus Genome Type**	**Number of Full-length Sequence Records**
Total virus genomes ^1^	27059 ^2^
dsDNA virus genomes	2419
ssDNA virus genomes	3607
dsRNA virus genomes	4861
ssRNA virus genomes	10665
ssRNA negative-strand virus genomes	1858
ssRNA positive-strand virus genomes	8801
unassigned/unclassified ssRNA genomes	6
Retro-transcribing virus genomess	4553
Deltavirus genomes	134
Satellite genomes	820
Virus genomes without classification	42

1 Only GenBank sequence records validated by RefSeq curators as full-length genomes are included.

2 Individual viral segments are included in tabulations, not complete constellations.
